# Donor-Acceptor Polymer Based on Planar Structure of Alkylidene-Fluorene Derivative: Correlation of Power Conversion Efficiency among Polymer and Various Acceptor Units

**DOI:** 10.3390/polym12122859

**Published:** 2020-11-29

**Authors:** Eui Jin Lee, Ho Jun Song

**Affiliations:** 1 Department of Materials Chemistry and Engineering, Konkuk University, 1 Hwayang-dong, Gwangjin-gu, Seoul 143-701, Korea; fkaus21c@naver.com; 2 Research Institute of Sustainable Manufacturing System, Intelligent Sustainable Materials R&D Group, Korea Institute of Industrial Technology, 89 Yangdaegiro-gil, Ipjang-myeon, Seobuk-gu, Cheonan-si 331-822, Chungcheongnam-do, Korea

**Keywords:** OPVs, conjugated polymer, alkylidene, quinoxaline

## Abstract

This study synthesized a novel polymer, poly(alkylidene fluorene-alt-diphenylquinoxaline) (PAFDQ), based on a planar alkylidene-fluorene and a highly soluble quinoxaline derivative through the Suzuki coupling reaction. We designed a novel molecular structure based on alkylidene fluorene and quinoxaline derivatives due to compact packing property by the planar structure of alkyidene fluorene and efficient intra-molecular charge transfer by quinoxaline derivatives. The polymer was largely dissolved in organic solvents, with a number average molecular weight and polydispersity index of 13.2 kg/mol and 2.74, respectively. PAFDQ showed higher thermal stability compared with the general fluorene structure owing to its rigid alkylidene-fluorene structure. The highest occupied and lowest unoccupied molecular orbital levels of PAFDQ were −5.37 eV and −3.42 eV, respectively. According to X-ray diffraction measurements, PAFDQ exhibited the formation of an ordered lamellar structure and conventional edge-on π-stacking. The device based on PAFDQ/Y6-BO-4Cl showed the best performance in terms of short circuit current (9.86 mA/cm^2^), open-circuit voltage (0.76 V), fill factor (44.23%), and power conversion efficiency (3.32%). Moreover, in the PAFDQ/Y6-BO-4Cl-based film, the phase separation of donor-rich and acceptor-rich phases, and the connected dark domains, was observed.

## 1. Introduction

Conducting molecules have long been utilized for diverse applications such as organic light-emitting diodes [1,2,3], organic solar cells (OSCs) [4,5,6,7,8,9,10,11,12,13,14,15], and organic thin-film transistors [16,17]. In particular, OSCs have received considerable attention because of the focus on economic viability and environmental friendliness. However, the low power conversion efficiency (PCE) of conjugated polymers has primarily hindered their use in OSCs [8]. Nevertheless, the donor-acceptor (D-A)-type conjugated polymer has received significant attention for several decades because its electronic characteristics can easily be tuned owing to the unique synergy between its donor and acceptor units. It can also extend its absorption range for wavelengths in the infrared region [8].

Ideally, in order to improve the efficiency of D-A-type conjugated polymers, the following characteristics are required [18]: (1) band gap property to absorb a wide range of wavelengths, (2) well-ordered orientation to exhibit a better charge transport property, (3) a low level of the highest occupied molecular orbital (HOMO) energy to show a high open-circuit voltage (V_OC_), and (4) an appropriate level of the lowest unoccupied molecular orbital (LUMO) energy for effective electron charge transfer to acceptor materials.

Better charge transport properties can be achieved by compact packing between the backbones of the polymer. This could lead to a decrease in the amorphous region by increasing the planar networks and efficient π-π stacking.

Fluorene derivatives exhibit good oxidation stability because of its low HOMO energy level [19]. Moreover, introducing two side chains at its 9-position improves the solubility of the fluorene derived conjugated polymers. However, fluorene has two alkyl side chains connected to the C atom with a “sticking-out” configuration. Therefore, it is difficult to form two-dimensional packing, which can lead to the suppression of compact packing and charge-carrier mobility [19,20]. Alkylidene fluorene instead of fluorene was reported by McCulloch et al. Its -lidene functional group is sp^2^-hybridized, which improves compact packing owing to the planar effect of the alkyl side chain.

In this study, we synthesized (D-A)-type polymers based on alkylidene fluorene and quinoxaline derivatives, and investigated their optical, surface, and morphological characteristics. In addition, their efficiency through optimized device environments (temperature, acceptor) was tested. We design a novel molecular structure based on alkylidene fluorene and quinoxaline derivatives due to compact packing property by the planar structure of alkylidene fluorene and efficient intra-molecular charge transfer by quinoxaline derivative.

## 2. Materials and Methods

### 2.1. Materials and Associated Methods

**Scheme 1:**
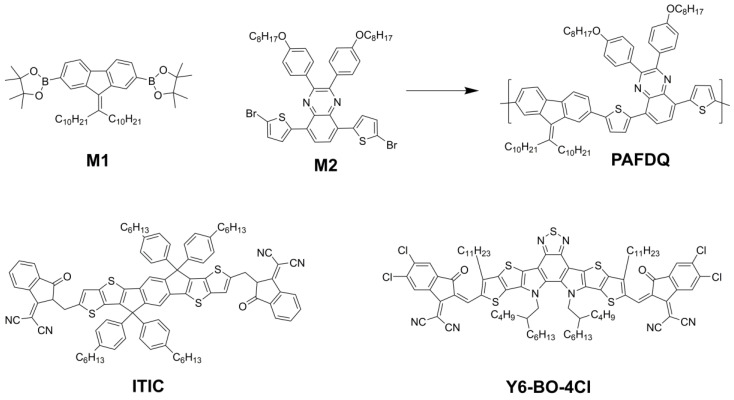
Monomer synthesis and polymerization.

All reagents were purchased from Aldrich, Acros, or TCI. All chemicals were used without further purification. The following compounds of Scheme 1 were synthesized following modified literature procedures: 2,7-Bis(4,4,5,5-tetramethyl-1,3,2-dioxaborolan-2-yl)-9-(1-decylundecylidene) fluorene [19].

5,8-bis(5-bromothiophen-2-yl)-2,3-bis(4-(octyloxy)phenyl)quinoxaline (**M2**) ^1^H NMR (400 MHz; CDCl_3_; Me_4_Si): 7.97(d, 2H;Ar) 7.63(d, 4H;Ar) 7.36(m, 6H;Ar) 7.14(d, 2H;Ar) 4.07(t, 4H;CH2) 1.83(m, 4H;CH2) 1.42(m, 4H;CH2) 1.30(m, 16H;CH2) 0.90(t, 6H;CH3). Anal. Calcd for: C_44_H_48_Br_2_N_2_O_2_S_2_: C, 61.39; H, 5.62; N, 3.25; O, 3.72; S, 7.45 Found: C, 61.23; H, 5.65; N, 3.13; O, 4.28; S, 7.42.

Poly(alkylidene fluorene-alt- diphenylquinoxaline) (**PAFDQ**), 5,8-bis(5-bromothiophen-2-yl)-2,3-bis(4-(octyloxy)phenyl)quinoxaline (M2) (0.27 g, 0.30 mmol), 2,7-bis(4,4,5,5-tetramethyl-1,3,2-dioxaborolan-2-yl)-9-(1-decylundecylidene) fluorene (M1) (0.33 g, 0.30 mmol), Pd(PPh_3_)_4_(0) (0.011 g, 0.01 mmol), and surfactant were placed in a Schlenk flask and purged with several cycles of nitrogen/vacuum cycles. Afterward, water-based 2 M K_2_CO_3_ (20 mL) and dry toluene (15 mL) were added. The reactant was heated to 100 °C and reacted in the dark for 24 h. After the reaction was complete, all the reactants were poured into methanol. The precipitate was filtered and purified by Soxhlet extraction with solvents in the following order: methanol, acetone, and chloroform. The final material was obtained as a red-violet solid (0.25 g, 55%). ^1^H NMR (CDCl_3_) d(ppm): 7.9–8.2 (br, 4H), 7.3–7.8 (br, 10H), 7.0–7.2 (br, 2H), 6.5–7.0 (br, 4H), 3.6–4.4 (br, 4H), 2.5–3.0 (br, 4H), 1.7–2.0 (br, 8H), 1.0–1.5 (br, 40H), 0.5–0.9 (br, 20H). Anal. calcd for: C_80_H_102_N_2_O_2_S_2_: C, 80.89; H, 8.66; N,2.36; O, 2.69; S, 5.40 found: C, 78.56; H, 7.99; N, 2.33; O, 3.31; S, 5.49.

### 2.2. Instruments and Characterization

All the reactions were performed under a nitrogen atmosphere. Organic solvents were dried by distillation. Column chromatography was conducted using silica gel (230-400 mesh, Merck). ^1^H-NMR spectra were measured with a Bruker ARX 400 spectrometer using solutions of CDCl_3_, and recorded in ppm units with tetramethylsilane as the standard. The elemental analyses were performed with an EA1112 using a CE Instrument. The absorption spectra were measured in an organic solvent with a Cintra 10e UV-Vis spectrophotometer. The cyclic voltammetric properties were measured with a DY2311 potentiostat/bipotentiostat (0.1 M acetonitrile solution including tetrabutyl ammonium hexafluorophosphate (Bu_4_NPF_6_) as the electrolyte) at a regular scan rate of 50 mV/s under a nitrogen condition. Patterned indium tin oxide (ITO), a Pt plate, and silver/silver chloride (Ag in 0.1 M KCl) were used as the working, counter, and reference electrodes, respectively. The HOMO levels of the polymers were determined using the oxidation onset value. Onset potentials are values obtained from the intersection of the two tangents drawn at the rising current and the baseline changing current of the CV curves. TGA measurements were conducted on the PerkinElmer TGA7. The molecular weight of the conjugated polymer was measured using gel permeation chromatography (GPC, Futecs, P-4000). All GPC analyses were made using tetrahydrofuran(THF) as eluant and polystyrene standard as reference. X-ray diffraction (XRD) patterns were obtained using SmartLab 3 kW (40 kV 30 mA, Cu target, wavelength: 1.541871 ang), Rigaku, Japan. The surface morphology of the blend layers was measured through atomic force microscopy (AFM) in the tapping mode using an XE-100 instrument. The photovoltaic performance of the OSCs was characterized under an AM 1.5 G condition of 100 mW/cm^2^ simulated sunlight by a solar cell measurement system (Oriel 96,000 150 W and Keithley 2400). The incident photon-to-current conversion efficiency (IPCE, McScience) was evaluated for the optimal device composition.

### 2.3. Fabrication of OSCs

To fabricate the OSC devices, ITO glass substrates were cleaned by sequential ultrasonic cleaning in detergent solution, 2-propanol, and deionized water. After cleaning, any moisture on the surface of the substrates was eliminated by exposure to N_2_ flow and a temperature of 120 °C. The dried substrates were transferred to the UV-ozone cleaner for surface treatment to improve interfacial contact. To fabricate inverted OSC devices, the ZnO precursor solution was deposited on top of the ITO surface by spin coating and annealed at 200 °C in the air to form an electron transport layer (~10 nm). The ZnO precursor solution was prepared according to the reported method [21]. The ZnO-coated ITO substrates were then transferred to a glove box filled with N_2_-gas, and a solution for the photoactive layer was deposited onto the ZnO layer by spin coating and annealed at 120 °C, for 10 min. The solutions were prepared by dissolving 10 mg of donor polymer in chlorobenzene with 10 mg of acceptors, respectively. (6,6)-phenyl C_71_ butyric acid methyl ester (PC_71_BM), 3,9-bis(2-methylene-(3-(1,1-dicyanomethylene)-indanone))-5,5,11,11-tetrakis(4-hexylphenyl)-dithieno(2,3-d:2′,3′-d’)-s-indaceno(1–b:5,6-b’)dithiophene (ITIC), and Y6-BO-4Cl were used as acceptors. Finally, MoO_3_ (5 nm) and Ag anodes (100 nm) were sequentially deposited by thermal evaporation in a high-vacuum chamber (<10^−7^ Torr). The active area of the fabricated inverted PSC was 4 mm^2^. We fabricated devices with more than 40 ea to confirm reproducibility.

## 3. Results and Discussion

### 3.1. Characterization of the Polymers

**Figure A1:**
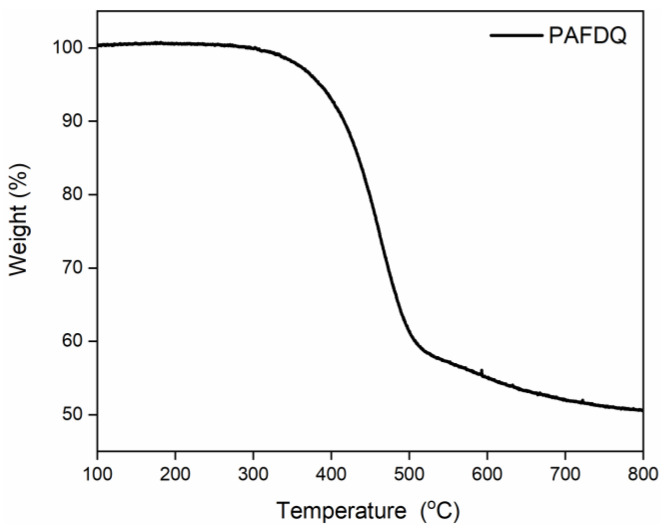
TGA data of PAFDQ.

**Figure A2:**
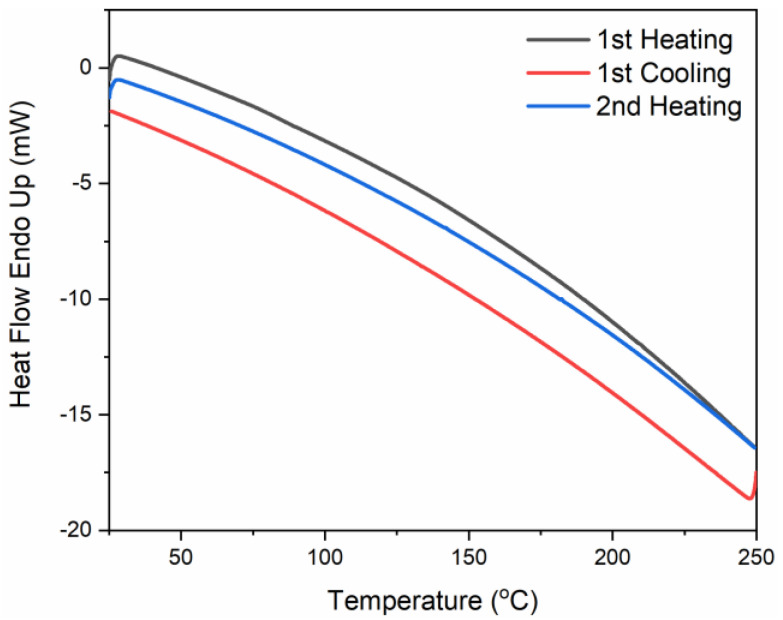
DSC curves of PAFDQ.

The molecular weight and thermal characteristics of PAFDQ are shown in Table 1. When GPC was performed using polystyrene as the standard, the number-average molecular weights (M_n_) and polydispersity index (PDI) of PAFDQ were 13,200 kg/mol and 2.74, respectively. The degree of polymerization in PAFDQ was lower than that of the general polymer, which may be due to the rigid alkylidene-fluorene derivative. TGA of PAFDQ showed a 5% weight loss at 385 °C (Table 1 & Figure A1), indicating higher thermal stability compared to the general donor structure, which can be attributed to the rigid alkylidene-fluorene structure. This high thermal stability makes the polymer applicable in various optoelectronic devices that require thermal stability over 300 °C [11]. We investigated the thermal behavior using DSC (see SI), which revealed no distinct thermal transitions in any of the polymers in the temperature range of 40–250 °C (Figure A2).

### 3.2. Optical and Electrochemical Properties

**Figure 1:**
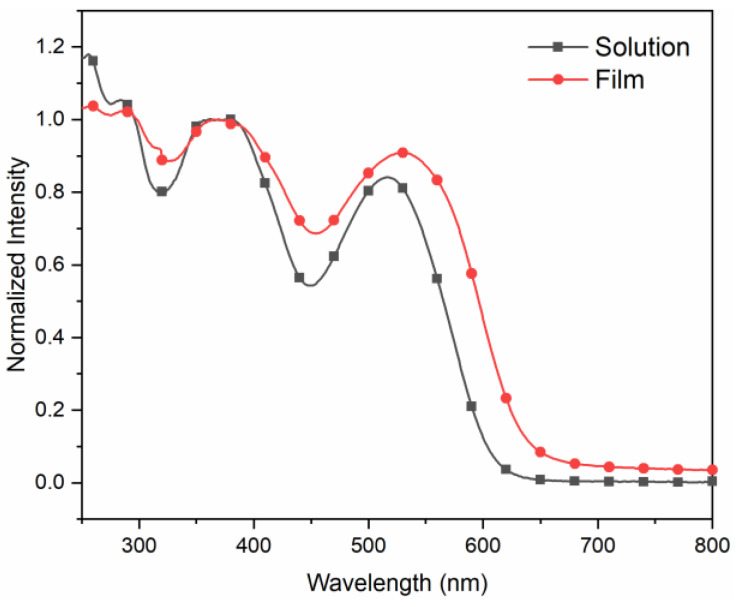
Absorption spectra of polymer in solution (10^−6^ M) film (50 nm).

The UV-visible (UV-vis) spectra of the polymer in solution and thin-film are exhibited in Figure 1. In solution, the maximum absorption peaks of PAFDQ (λ_max_) were observed at 256, 284, 362, and 516 nm. The band near the 300–400 nm range occurred due to the π-π* transition, whereas the one at 530–600 nm occurred due to ICT between the donor and acceptor moieties [12]. The maximum absorption spectra of the polymer in the film were red-shifted compared to those in the solution. In the thin film, the maximum absorption peaks of PAFDQ (λ_max_) were observed at 259, 286, 365, and 529 nm, caused by their more-planar organizations in the solid-state. The optical band gap of PAFDQ calculated through the UV onset value of the film was 1.95 eV. The PAFDQ showed less red-shift spectrum(529 nm) compared with quinacridone-quinoxaline-based polymer (538 nm), which means the effective intramolecular charge transfer has not occurred well in PAFDQ backbone than quinacridone-quinoxaline backbone [11,14].

**Figure 2:**
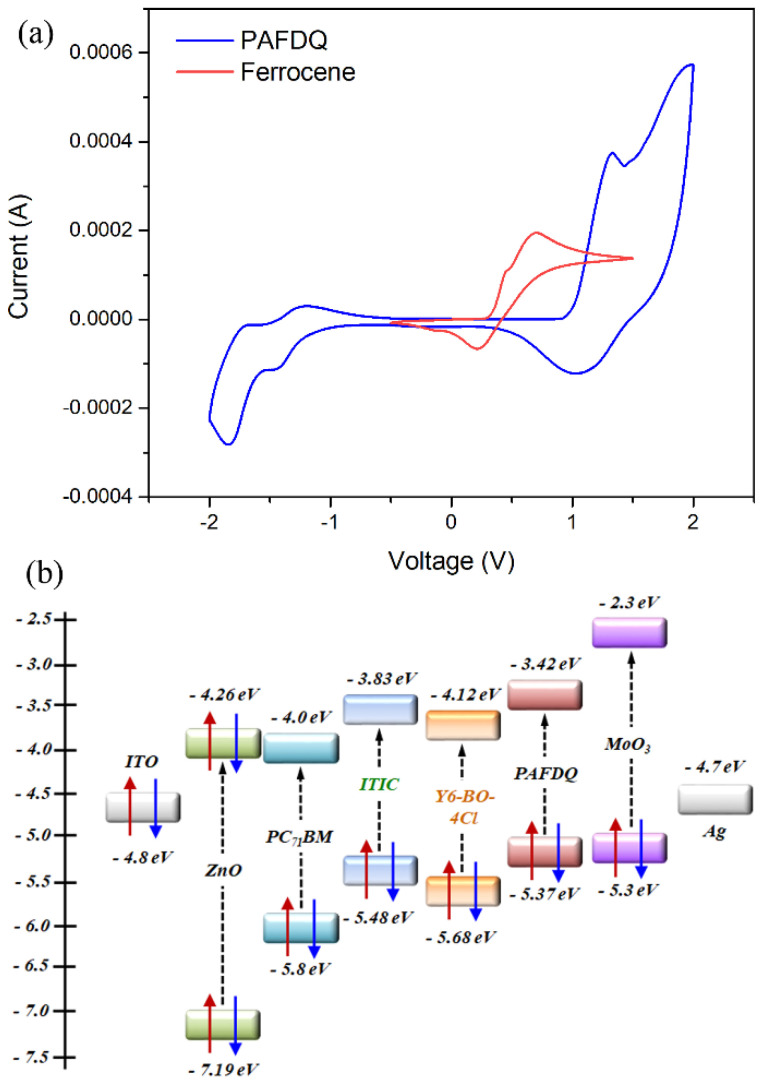
(**a**) Cyclic voltammogram of polymers with a 0.1 M acetonitrile (substituted with nitrogen for 5 min) solution (**b**) band diagram of polymer (experimental data), ITO, ZnO, PC_71_BM, ITIC, Y6-BO−4Cl, MoO_3_, and Ag. (The energy levels of ITO, ZnO, PC_71_BM, MoO_3_, Ag [21], ITIC [22], and Y6-BO-4Cl [23] were obtained from reported literature).

Figure 2a shows the cyclic voltammograms of PAFDQ, where a distinct oxidation-reduction peak was observed in PAFDQ because it is D-A structured, unlike poly(quinacridone-bithiophene) (PQA2T), which is of the typical p-type structure [16]. The optical and electrochemical properties of the polymers are summarized in Table 2. The oxidations (E_ox_^onset^) of PAFDQ were +0.99 V, and the HOMO level was calculated to be −5.37 eV. It is expected that PAFDQ exhibits high air stability and V_OC_ owing to low HOMO levels. The LUMO energy level was calculated from the difference between the HOMO energy levels and optical band gap energies, yielding −3.42 eV.

Figure 2b presents the band diagram of the energy levels obtained through cyclic voltammetry (CV). The LUMO level of PAFDQ (−3.42 eV) was lower than that of PTB7 (−3.31 eV) by 0.11 eV. Therefore, if charge separation occurred after receiving light energy, an electron in PAFDQ would exhibit more effective charge transport to PCBM than an electron in PTB7 in the active layer [18].

### 3.3. Orientation Analysis

**Figure 3:**
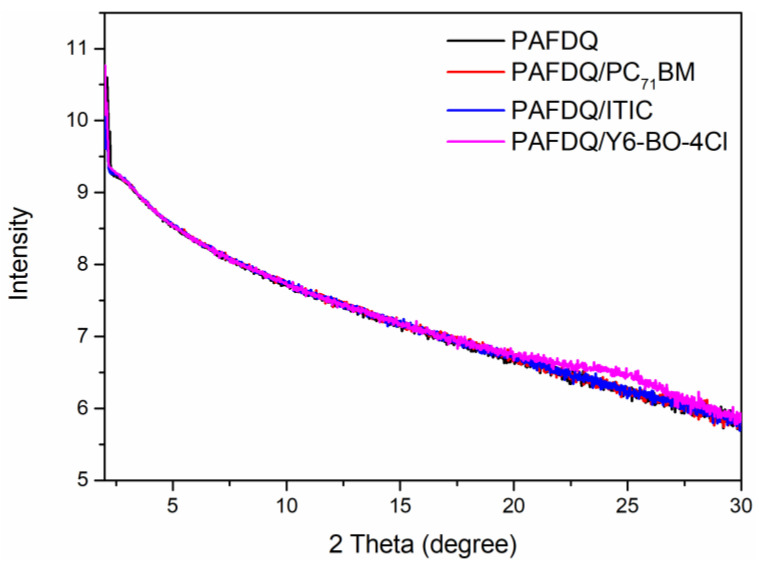
Out-of-plane in X-ray diffraction pattern in thin films after thermal treatment.

Figure 3 presents the XRD measurements to analyze the order of the structure of PAFDQ and various other acceptors. PAFDQ exhibited sharp diffraction peaks at 2.1 and 2.2° in the out-of-plane peak (100), indicating the formation of an ordered lamellar structure by the alkyl side chain of alkylidene-fluorene and conventional edge-on π-stacking [11,16]. The d_π_-spacing with only PAFDQ was 4.0–4.2 nm, whereas the (010) diffraction peak corresponding to the π-π stacking rarely appeared. Similarly, sharp diffraction peaks in the out-of-plane peak were observed for the PAFDQ/ PC_71_BM and PAFDQ/ ITIC films. However, the PAFDQ/Y6-BO-4Cl film exhibited a more prominent (010) diffraction peak corresponding to the π-π stacking in the out-of-plane pattern at 23.0–27.0°. The π-π stacking distance was 3.30–3.86 Å. This suggests that a large fraction of the PAFDQ/Y6-BO-4Cl film was oriented face-on relative to the substrate due to the interaction between the PAFDQ and Y6-BO-4Cl derivatives. The PAFDQ/Y6-BO-4Cl film exhibited the strongest π-π stacking interaction and face-on orientation compared to other films, which is expected because it exhibited the best fill factor (FF) by a low series resistance [11].

### 3.4. Photovoltaic Characteristics

**Figure 4:**
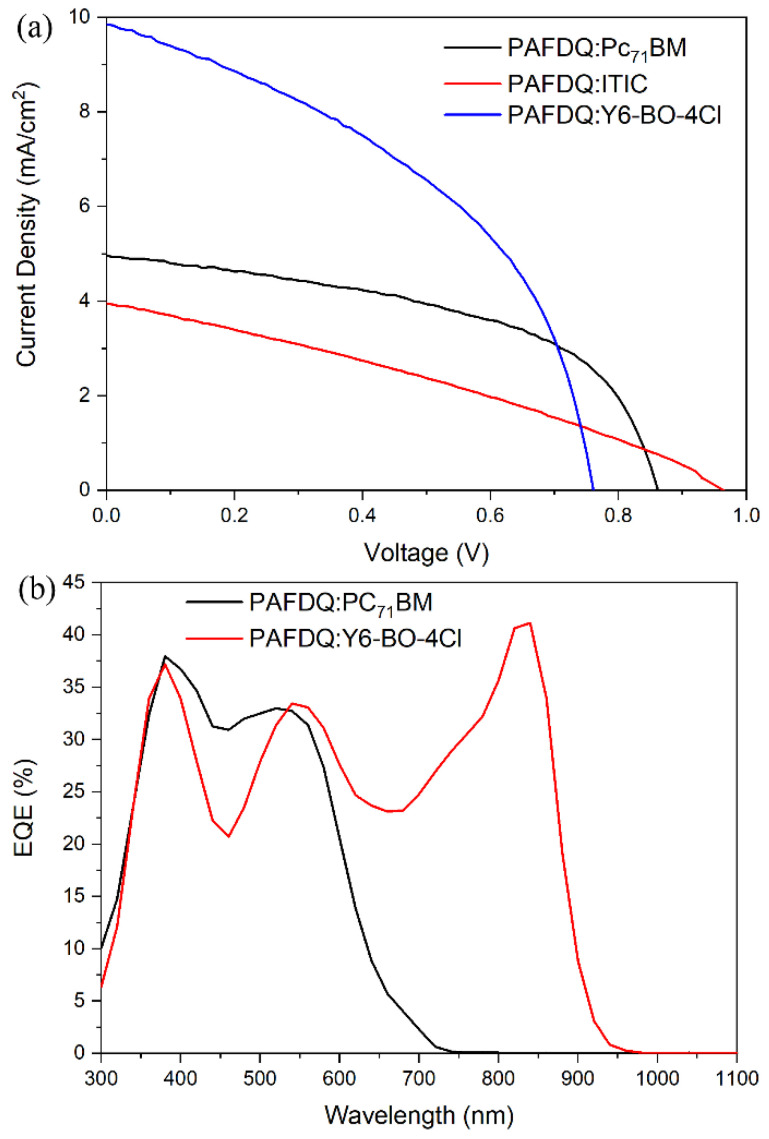
Photovoltaic performance of organic solar cells (OSCs). (**a**) *J-V* curves and (**b**) external quantum efficiency (EQE) spectra.

Figure 4 and Table 2 show the evaluation results of the OSCs. The active layer had an optimized blending ratio obtained by dissolving the PAFDQ polymer and acceptors (PC_71_BM, ITIC, or Y6-BO-4Cl) in chlorobenzene using a concentration of approximately 0.5–1 wt %. The spin-cast film was annealed at 120°C for 10 min. The device based on the non-fullerene acceptor (NFA) of Y6-BO-4Cl showed the best performance in terms of short circuit current (J_SC_) = 9.86 mA/cm^2^, V_OC_ = 0.76 V, FF = 44.23%, and PCE = 3.32%. It is noteworthy that the J_SC_ value was twice that of the PCBM-based device. This can be attributed to the wider absorption wavelength band of Y6-BO-4Cl/NFA. As observed in the IPCE data (Figure 4b), the device with Y6-BO-4Cl NFA exhibited a higher external quantum efficiency (EQE) and wider absorption band than that of the PC_71_BM-based device.

### 3.5. Morphology Analysis

**Figure 5:**
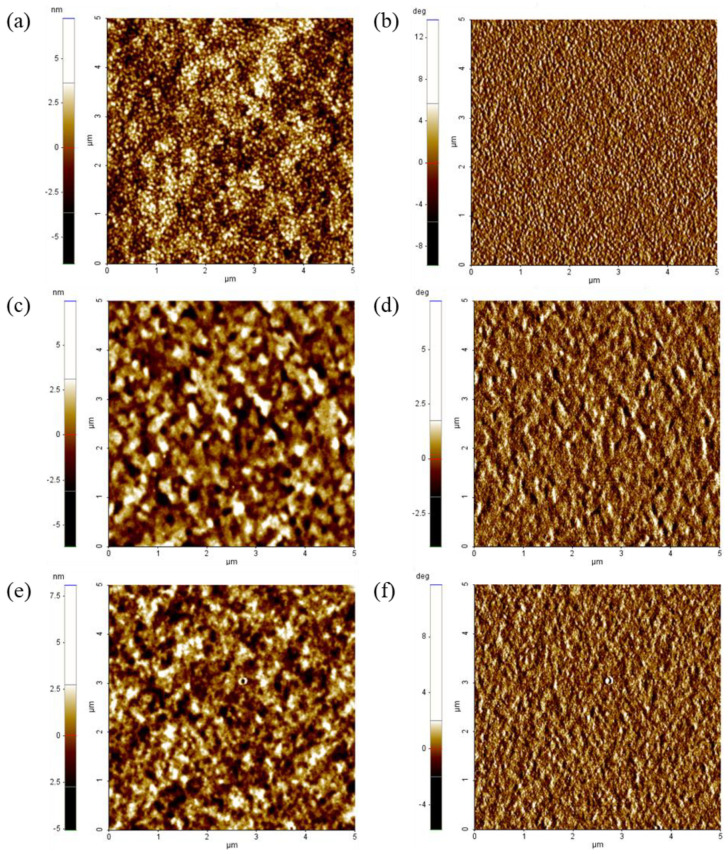
Atomic force microscopy (AFM) topographic and phase images of BHJ films (**a**,**b**) PAFDQ:PC_71_BM (R_RMS_ = 1.77 nm), (**c**,**d**) PAFDQ:ITIC (R_RMS_ = 1.51 nm), (**e**,**f**) PAFDQ: Y6-BO-4Cl (R_RMS_ = 1.34 nm).

AFM results show large aggregates in the polymer/NFA acceptor films with a smaller root-mean-square roughness (R_RMS_) (Figure 5). In the Y6-BO-4Cl based film (Figure 5e,f), the phase separation of donor-rich (bright domains) and acceptor-rich (dark domains) phases, and connected dark domains was observed. However, in the ITIC-blended film (Figure 5c,d), larger domains were observed without their interconnection. The connected domains can form a charge transport channel, which can improve charge extraction and suppress charge recombination. [24,25]. By using ITIC as the acceptor, higher V_OC_ can be achieved because of the smaller HOMO level offset with the donor polymer, when compared to Y6-BO-4Cl [24,25,26]. However, better phase separation occurred in the Y6-BO-4Cl-blended active layer than in the ITIC-blended film, resulting in higher J_SC_ and FF in the Y6-BO-4Cl-based devices.

## 4. Conclusions

In this study, an alkylidene-fluorene-quinoxaline-based polymer was successfully synthesized using the Suzuki coupling reaction by introducing a planar alkylidene-fluorene unit and a highly soluble quinoxaline unit. The polymer exhibited high molecular weights and good thermal stability. According to XRD measurements, PAFDQ exhibited a formation of an ordered lamellar structure and conventional edge-on π-stacking. The device based on PAFDQ/Y6-BO-4Cl showed the best performance in terms of *J_SC_* = 9.86 mA/cm^2^, *V_OC_* = 0.76 V, PCE = 3.32%, and *FF* = 44.23%, with the FF being higher than that of PAFDQ/ITC (31.56). In addition, the phase separation of donor-rich and acceptor-rich phases, and connected dark domains was observed in the PAFDQ/Y6-BO-4Cl film.

## Figures and Tables

**Table 1:** Molecular weight and thermal properties and Optical/electrochemical properties of the polymers.

Polymer	*M*_n_ (kg/mol)	*M*_w_ (kg/mol)	PDI	T*_d_* (°C)	Absorption, λ_max_ (nm)		*E*_ox_^onset^ (V)	*E*_HOMO_ (eV) *^a^*	*E*_LUMO_ (eV) *^b^*	*E*_opt_ (eV) *^e^*
					Solution *^a^*	Film *^b^*				
PAFDQ	13,200	36,400	2.74	385	256, 284, 362, 516	259, 286, 365, 529	0.99	−5.37	−3.42	1.95

*^a^* Calculated from the oxidation onset potentials under the assumption that the absolute energy level of Fc/Fc+ was −4.8 eV below a vacuum. *^b^* HOMO—Eopt. *^e^* Estimated from the onset of UV-vis absorption data of the thin film.

**Table 2:** Photovoltaic performance of the OSCs.

Active Layer	Weight Ratio (P:A, *w*/*w*)	V_OC_ (V)	J_SC_ (mA/cm^2^)	FF (%)	PCE (%)
Polymer: Acceptor					
PAFDQ: PC_71_BM	1:2	0.86	4.97	51.63	2.21
	average	0.85 ± 0.02	4.83 ± 0.09	51.66 ± 0.64	2.11 ± 0.06
PAFDQ: ITIC	1:1	0.96	3.94	31.56	1.20
	average	0.93 ± 0.02	3.77 ± 0.12	32.10 ± 1.63	1.12 ± 0.06
PAFDQ: Y6-BO-4Cl	1:1	0.76	9.86	44.23	3.32
	average	0.75 ± 0.01	9.63 ± 0.34	44.20 ± 1.52	3.18 ± 0.11

## References

[1] Friend R.H., Gymer R.W., Holmes A.B., Burroughes J.H., Marks R.N., Taliani C., Bradley D.D.C., Dos Santos D.A., Brédas J.L., Lögdlund M. (1999). Electroluminescence in conjugated polymers. Nature.

[2] Breen C.A., Tischler J.R., Bulović V., Swager T.M. (2005). Highly efficient blue electroluminescence from poly(phenylene ethynylene) via energy transfer from a hole-transport matrix. Adv. Mater..

[3] Song H.J., Lee J.Y., Song I.S., Moon D.K., Haw J.R. (2011). Synthesis and electroluminescence properties of fluorene-anthracene based copolymers for blue and white emitting diodes. J. Ind. Eng. Chem..

[4] Krebs F.C. (2009). Polymer solar cell modules prepared using roll-to-roll methods: Knife-over-edge coating, slot-die coating and screen printing. Sol. Energy Mater. Sol. Cells.

[5] Subramaniyan S., Xin H., Kim F.S., Shoaee S., Durrant J.R., Jenekhe S.A. (2011). Effects of side chains on thiazolothiazole-based copolymer semiconductors for high performance solar cells. Adv. Energy Mater..

[6] Cheng P., Wang H., Zhu Y., Zheng R., Li T., Chen C., Huang T., Zhao Y., Wang R., Meng D. (2020). Transparent Hole-Transporting Frameworks: A Unique Strategy to Design High-Performance Semitransparent Organic Photovoltaics. Adv. Mater..

[7] Cheng P., Wang J., Zhan X., Yang Y. (2020). Constructing High-Performance Organic Photovoltaics via Emerging Non-Fullerene Acceptors and Tandem-Junction Structure. Adv. Energy Mater..

[8] Kim D.H., Seok W.C., Leem J.T., Han Y.W., Kang J.H., Song H.J. (2019). Ordered orientation and compact molecule packing due to coplanar backbone structure of interlayer: Improvement in fill factor for photovoltaic device. Eur. Polym. J..

[9] Manceau M., Angmo D., Jørgensen M., Krebs F.C. (2011). ITO-free flexible polymer solar cells: From small model devices to roll-to-roll processed large modules. Org. Electron..

[10] Song H.J., Kim D.H., Lee E.J., Moon D.K. (2013). Conjugated polymers consisting of quinacridone and quinoxaline as donor materials for organic photovoltaics: Orientation and charge transfer properties of polymers formed by phenyl structures with a quinoxaline derivative. J. Mater. Chem. A.

[11] Song H.-J., Kim D.-H., Lee E.-J., Heo S.-W., Lee J.-Y., Moon D.-K. (2012). Conjugated Polymer Consisting of Quinacridone and Benzothiadiazole as Donor Materials for Organic Photovoltaics: Coplanar Property of Polymer Backbone. Macromolecules.

[12] Zhu Y., Champion R.D., Jenekhe S.A. (2006). Conjugated donor-acceptor copolymer semiconductors with large intramolecular charge transfer: Synthesis, optical properties, electrochemistry, and field effect carrier mobility of thienopyrazine-based copolymers. Macromolecules.

[13] Kuwabara J., Nohara Y., Choi S.J., Fujinami Y., Lu W., Yoshimura K., Oguma J., Suenobu K., Kanbara T. (2013). Direct arylation polycondensation for the synthesis of bithiophene-based alternating copolymers. Polym. Chem..

[14] Song H.J., Kim D.H., Lee E.J., Haw J.R., Moon D.K. (2014). Correlation of intramolecular charge transfer and orientation properties among quinacridone and acceptor units. Sol. Energy Mater. Sol. Cells.

[15] Cheng P., Wang H., Zheng R., Zhu Y., Dai S., Li Z., Chen C., Zhao Y., Wang R., Meng D. (2020). Enabling High-Performance Tandem Organic Photovoltaic Cells by Balancing the Front and Rear Subcells. Adv. Mater..

[16] Osaka I., Akita M., Koganezawa T., Takimiya K. (2012). Quinacridone-based semiconducting polymers: Implication of electronic structure and orientational order for charge transport property. Chem. Mater..

[17] Lin C.J., Lee W.Y., Lu C., Lin H.W., Chen W.C. (2011). Biaxially extended thiophene-fused thiophene conjugated copolymers for high performance field effect transistors. Macromolecules.

[18] Jiang J.M., Yang P.A., Hsieh T.H., Wei K.H. (2011). Crystalline low-band gap polymers comprising thiophene and 2,1,3-benzooxadiazole units for bulk heterojunction solar cells. Macromolecules.

[19] Song K.W., Song H.J., Lee T.H., Heo S.W., Moon D.K. (2013). An effect on the side chain position of D-π-A-type conjugated polymers with sp2-hybridized orbitals for organic photovoltaics. Polym. Chem..

[20] Liu J., Choi H., Kim J.Y., Bailey C., Durstock M., Dai L. (2012). Highly crystalline and low bandgap donor polymers for efficient polymer solar cells. Adv. Mater..

[21] Lee E.J., Heo S.W., Han Y.W., Moon D.K. (2016). An organic–inorganic hybrid interlayer for improved electron extraction in inverted polymer solar cells. J. Mater. Chem. C.

[22] Lin Y., Wang J., Zhang Z.-G., Bai H., Li Y., Zhu D., Zhan X. (2015). An Electron Acceptor Challenging Fullerenes for Efficient Polymer Solar Cells. Adv. Mater..

[23] Cui Y., Yao H., Zhang J., Zhang T., Wang Y., Hong L., Xian K., Xu B., Zhang S., Peng J. (2019). Over 16% efficiency organic photovoltaic cells enabled by a chlorinated acceptor with increased open-circuit voltages. Nat. Commun..

[24] Yu R., Yao H., Cui Y., Hong L., He C., Hou J. (2019). Improved Charge Transport and Reduced Nonradiative Energy Loss Enable over 16% Efficiency in Ternary Polymer Solar Cells. Adv. Mater..

[25] Chang Y., Lau T.K., Pan M.A., Lu X., Yan H., Zhan C. (2019). The synergy of host-guest nonfullerene acceptors enables 16%-efficiency polymer solar cells with increased open-circuit voltage and fill-factor. Mater. Horizons.

[26] Coropceanu V., Chen X.K., Wang T., Zheng Z., Brédas J.L. (2019). Charge-transfer electronic states in organic solar cells. Nat. Rev. Mater..

